# Composites Filled with Metal Organic Frameworks and Their Derivatives: Recent Developments in Flame Retardants

**DOI:** 10.3390/polym14235279

**Published:** 2022-12-02

**Authors:** Ping Lyu, Yongbo Hou, Jinhu Hu, Yanyan Liu, Lingling Zhao, Chao Feng, Yong Ma, Qin Wang, Rui Zhang, Weibo Huang, Mingliang Ma

**Affiliations:** 1 School of Civil Engineering, Qingdao University of Technology, Qingdao 266520, China; 2 School of Material Science and Engineering, Shandong University of Science and Technology, Qingdao 266590, China

**Keywords:** metal organic frameworks (MOFs), polymers, hybrids, fire retardancy, flame-retardant mechanism

## Abstract

Polymer matrix is vulnerable to fire hazards and needs to add flame retardants to enhance its performance and make its application scenarios more extensive. At this stage, it is more necessary to add multiple flame-retardant elements and build a multi-component synergistic system. Metal organic frameworks (MOFs) have been studied for nearly three decades since their introduction. MOFs are known for their structural advantages but have only been applied to flame-retardant polymers for a relatively short period of time. In this paper, we review the development of MOFs utilized as flame retardants and analyze the flame-retardant mechanisms in the gas phase and condensed phase from the original MOF materials, modified MOF composites, and MOF-derived composites as flame retardants, respectively. The effects of carbon-based materials, phosphorus-based materials, nitrogen-based materials, and biomass on the flame-retardant properties of polymers are discussed in the context of MOFs. The construction of MOF multi-structured flame retardants is also introduced, and a variety of MOF-based flame retardants with different morphologies are shown to broaden the ideas for subsequent research.

## 1. Introduction

Fire is the beacon that lights up human life, and fire is a major hidden danger that threatens natural society and human life. Fire protection is not limited to traditional flammable materials such as wood, paper, and cotton, but also to new polymer materials. With the advantages of wear resistance, corrosion resistance, and good insulation, traditional steel products have been replaced by polymers in some fields. However, they display a large fire load and release a significant amount of heat and smoke when burning, which will cause property damage and personal injury [1,2]. Polymer flame retardants have been researched, but with them comes the possibility of environmental pollution [3,4,5,6]. In line with the green and environmental protection advocated at this stage, high-efficiency and environment-friendly flame retardants are bound to become a hot spot for research. The functionalization of metal organic frameworks (MOFs) has therefore drawn attention [7].

MOFs are coordination networks with organic ligands containing potential voids [8]. Due to their structural diversity and tunability, they continue to be widely used, including in, but not limited to, high-performance sensing materials [9,10], high-activity catalyst development [11,12], supercapacitors [13,14], microwave absorption [15,16], drug delivery [17], luminescent materials, nanomaterial carriers, polymerization, and imaging [18]. Tens of thousands of MOFs have been designed and synthesized since MOFs were explicitly proposed in the 1990s [19]. At the same time, MOF-based hybrids and MOF-derived materials are emerging one after another, which also provides a variety of strategies and approaches to better utilize the properties of MOF structures [20,21]. MOFs, also known as porous coordination networks (PCNs), have a robust structure that can generate permanently stable pores with tunable and controllable properties [22]. In connection with the fact that the smoke released when the polymer is burned is the most deadly hazard to humans in a fire, the rich mesh structure can delay the release of smoke [23]. Therefore, the porous structure of MOFs provides potential for their use as flame retardants. Smoke release retardation is good, but flame retardants can further inhibit heat transfer and smoke release by generating a good residual as a barrier, directly effectively reducing the release with high quality and abundant amounts of residual char. There have been some reports that transition metals can catalyze carbonization and reduce the rate of heat release [24,25]. Transition metals could form C-C bonds during combustion, which helps to convert the polymer matrix into char with considerable stability, thereby improving the flame-retardant efficiency. On the one hand, the above transition metal elements can just fit the metal nodes of MOFs, and various MOF structures also prompt researchers to have more choices for metal sites. On the other hand, the presence of organic ligands, another important component of MOFs, can provide partial flame-retardant elements and groups, while also enhancing compatibility with the polymer matrix. Hou et al. proposed possible flame-retardant mechanisms for organic ligands: condensed-phase catalysis into carbon, increased barrier effect, gas-phase dilution of combustible gases, and quenching of free radicals [26]. It is the mutual agreement of the two aspects that makes MOF-based flame retardants combined with the advantages of organic and inorganic flame retardants full of more possibilities. In addition, superior performance flame retardants may also be accompanied by excellent thermal stability, high limiting oxygen index (LOI), and low dripping.

**Figure 1:**
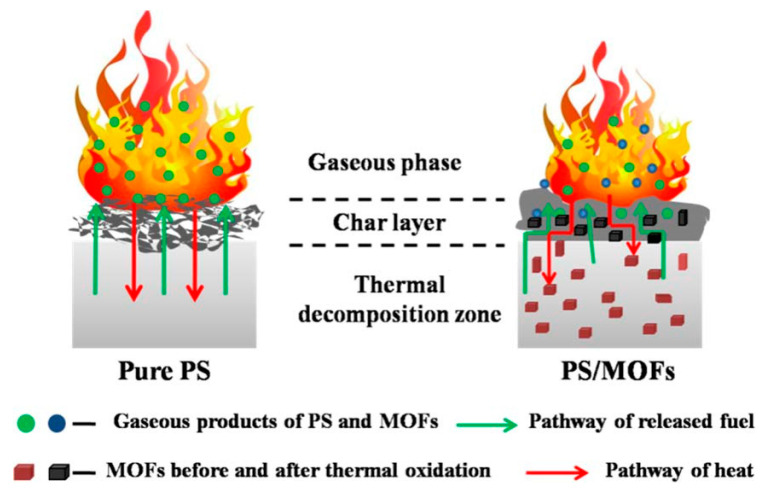
Proposed mechanisms for illustration of the thermal stability flame retardancy of PS and its composites. Reprinted with permission from Ref. [27]. Copyright 2017, American Chemical Society.

In 2017, the addition of MOFs to polystyrene (PS) as flame retardants was first reported by Hu et al. [27]. The synthesized iron-based and cobalt-based MOFs could significantly improve the flame-retardant properties of PS composites, and the corresponding MOF’s flame-retardant mechanisms were proposed: thermal barrier effect and catalysis. During the pyrolysis of the PS matrix, the gaseous products of MOFs reduce the concentration of degradation products and reactive oxygen species, whereas the porous metal oxides act as heat and fuel transfer barriers. The flame-retardant mechanism is shown in Figure 1.

**Figure 2:**
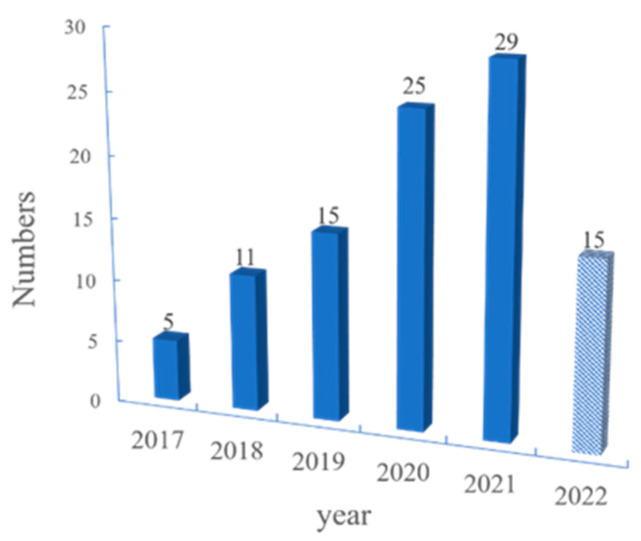
Number of publications vs. year for metal organic frameworks and their derivatives as flame retardants (until June 2022).

Since then, researchers have carried out a significant amount of research on the flame retardancy of MOFs, and pristine MOFs are naturally the primary target of research. Therefore, MOF derivatives, combined with phosphorus and nitrogen compounds or carbon-based materials, have been explored in greater depth. This work organizes the above categories and organizes the number of publications about MOF flame retardants in each year. It can also be confirmed from Figure 2 that research regarding MOF flame retardants is increasing each year, indicating that they have received more and more attention. It is hoped that this review can provide some reference and guidance for future research related to flame retardancy in MOFs based on the classification.

## 2. Pristine MOFs Directly as Flame Retardants

**Figure 3:**
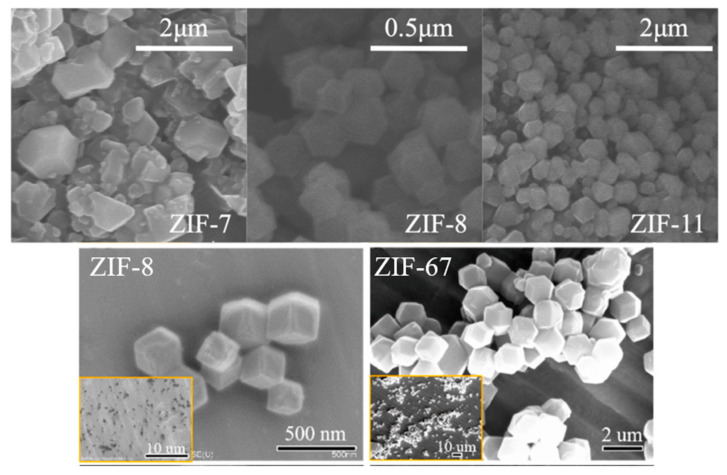
SEM images of ZIF-7, ZIF-8, and ZIF-11 (**above**) and ZIF-8 and ZIF-67 (**below**). Reprinted with permission from Ref. [26]. Copyright 2021 Elsevier and Ref. [34].

Because some of the MOFs whose own metal sites belong to transition metal elements (often Zn, Co, Zr, etc.) have catalytic carbonization properties, pristine MOF monomers can be added directly to the polymer matrix as flame retardants. During combustion, the metal sites of MOFs generate metal oxides that promote the formation of a continuous dense char layer to insulate part of the combustible gases and heat [28]. Shi et al. added synthetic zeolitic imidazolate framework-8 (ZIF-8) nanoparticles directly into poly (lactic acid) (PLA), and an addition of 0.2 wt% could increase the limiting oxygen index value from 20.5% to 23.5% [29]. Similarly, on polyvinyl alcohol aerogel (PVA) [30], flexible polyurethane foam (MFPU) [31], and even PLA non-woven fabric [32], the LOI has been significantly improved after adding ZIF monomer. Among them, when 1:1 ZIF-8 was added to the PVA aerogel, the peak heat release rate (pHRR) was significantly reduced by 44.2%. ZIFs had synthetic and structural similarities, and some of their microscopic morphologies are shown in Figure 3. The properties of ZIFs applied in flame retardants may also show similarity, as can be demonstrated by the performance tests of ZIF composites synthesized by some researchers [26,33,34]. Considering the excellent thermal stability and mechanical properties of zirconium-based MOFs [35,36], Sai et al. [37] directly blended UiO-66 with different mass fractions with polycarbonate (PC). The samples with 4 wt% UiO-66 added could reach the V-0 level. Time to ignite (TTI) and t-pHRR (time to reach pHRR) were delayed by 90 and 75 s, respectively, which would provide significantly more time for help for personnel rescue in real fires. Chen et al. [38] added UiO-66 directly to PS, and the smoke suppression effect was obvious. The total smoke release (TSR) of the PS/UiO-66-5 wt% composite was reduced by 35% compared with pure PS. In addition, compared to the large amount of dripping and black smoke from pure PS, the PS/UiO-66 composite does not have a similar situation because the heat-resistant material ZrO2 is generated during the reaction and acts as a good barrier.

**Figure 4:**
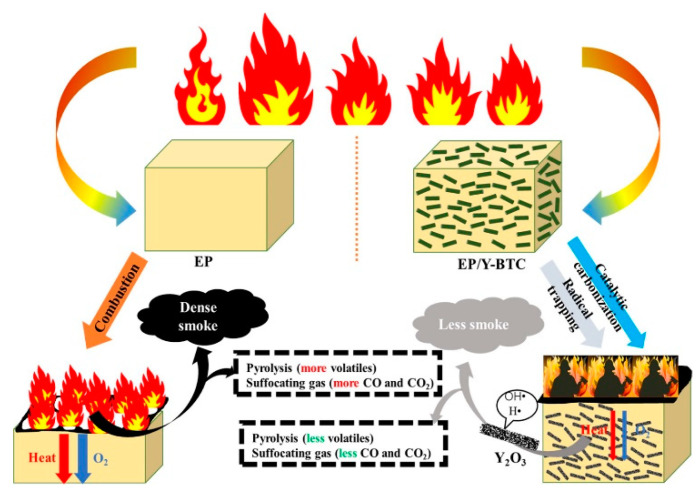
Schematic illustration of the proposed mechanism of EP/Y-BTC. Reprinted with permission from Ref. [44]. Copyright 2021, American Chemical Society.

To explore the different metals used as flame-retardant materials for unmodified MOFs, several MOFs, including Fe [26], Cr [39], Cu [40], and rare earth elements have been synthesized and blended directly into a polymer matrix to investigate their thermal stability. Mo-MOF was prepared and incorporated into epoxy (EP) by Zhang et al. [41]. Probably due to its good dispersion state and compatibility, the appropriate amount of Mo-MOF helped to enhance the mechanical properties of EP. The EP composites with the addition of 6 wt% Mo-MOF could reduce the pHRR and peak CO production (PCOP) by 44.7% and 42.9%, respectively. Some rare earth elements have thermal stability and toxic fume inhibition, and can be tried as flame retardants [42,43]. Li et al. [44] investigated three rare earth elements (La, Ce, and Y) as metal sites for MOFs (RE-MOF). The EP composites were tested for flame retardancy using the cone calorimeter test (CCT) while comparing the reasons for the high and low flame retardancy of the three RE-MOF. The best performing Y-MOF has excellent catalytic charring and radical trapping ability, thus blocking the EP degradation process, and the possible flame-retardant mechanism is shown in Figure 4. Specifically, Y can catalyze the formation of carbon layers possessing a higher degree of graphitization, and the formed Y_2_O_3_ can trap the free radicals in the chain reaction.

The direct blending of some of the initial MOFs into a polymer matrix has demonstrated the indispensable ability of MOF materials for flame-retardant applications. However, because the gains obtained are from their own structure, they partly require higher additions while causing a loss of mechanical properties. If people want to further improve the fire performance of polymers, it is inevitable to consider combining them with other materials.

## 3. Hybrid of Phosphorus–Nitrogen Compounds and MOFs as Flame Retardants

**Figure 5:**
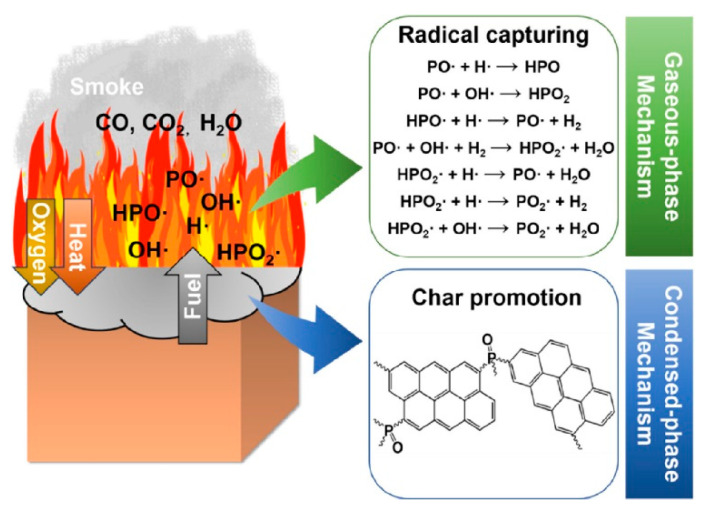
The flame-retardant mechanism of P-containing flame retardants: radical capturing effect in the gaseous phase and char promotion effect in the condensed phase. Reprinted with permission from Ref. [51]. Copyright 2021, Elsevier.

Phosphorus and nitrogen elements belong to the same group of elements, and both belong to the common non-halogen flame-retardant elements. At the same time, because halogens are regarded as substances that are toxic and affect environmental permanence, halogen-free flame retardants will naturally receive more attention [45,46,47]. Traditional phosphorus-based flame retardants include red phosphorus, phosphate, ammonium polyphosphate (APP), etc., while nitrogen-based flame retardants contain melamine and dicyandiamide [48,49,50,51]. Because phosphorus-based flame retardants and nitrogen-containing compounds are found to have a good synergistic effect, the two elements are also often combined in the design of flame retardants to bring into play the P-N synergistic effect. On the one hand, phosphorus flame retardants can release PO· and PO_2_· radicals and other reactive radicals such as H·, HO·, and O· generated in the combustion chain reaction burst reaction. On the other hand, phosphorus flame retardants can release some phosphoric acid-containing to promote the dehydration of polymer substrates into carbon, as shown in Figure 5. As the mechanism diagram shows, most conventional phosphorus flame retardants have a single flame-retardant mechanism, while P-N flame retardants can effectively improve the thermal stability and fire safety performance of polymers [52]. The compatibility exhibited by inorganic flame-retardant fillers with polymers is poor, while MOFs can form mutual attraction with polymer chains to improve the compatibility. The metal oxides generated from MOFs during pyrolysis also further promote catalytic carbonization [53].

### 3.1. Ammonium Salts and MOFs as Flame Retardants

In recent years, ammonium salt flame retardants have attracted attention due to their ease of preparation, clean production, application and disposal with less harm to the environment; they mainly include APP, melamine phosphate (MP), and melamine polyphosphate (MPP). APP, also known as ammonium polyphosphate, is used quite widely as the main type of non-halogen flame retardant. However, due to its limited flam- retardant effect alone, it is often used in combination with other halogen-free flame retardants. When used in combination, they can not only reduce the amount of APP but also improve the flame retardants efficiency. At the same time, compound flame retardants can reduce the loss of mechanical properties [54]. Chen et al. [55] first prepared Cu-based MOF materials and then used the Cu-MOF and APP mass fractions added as controls according to the differences. The effect of physical mixing of MOF materials with APP as a flame retardant for thermoplastic polyurethane (TPU) has been investigated. CCT and LOI tests showed that the flame-retardant property of TPU composites was further enhanced when Cu-MOF was used in combination with APP. When Cu-MOF was added, only 0.0625 wt%, the LOI level reached 28%, and the pHRR and total heat release (THR) values were reduced by 19.2% and 63.4% compared with TPU/APP. Table 1 also summarizes the currently reported data of MOF/APP synergistic flame retardants, as well as the combustion data of pure polymer matrix and no MOFs. Through the data analysis in Table 1, it can be confirmed that adding a certain amount of MOFs compared to only adding APP as a flame retardant shows a trend of improving the flame-retardant performance. However, the improved effect is different, which may be caused by the different types of substrates and MOFs. In addition to physical mixing, Wang et al. [56] assembled ZIF-67 onto the APP surface, which opened new avenues for MOFs while obtaining excellent flame-retardant properties. The synthesized APPZ4 was added to Ethylene-vinyl acetate (EVA) as a flame-retardant composite, and with the increase of temperature, APPZ4 could be converted to cobalt phosphate nanocatalyst and formed di-micro nano-films and aggregated nano-skeletons. It exerts catalytic graphitization on the surface of the EVA matrix and generates a large amount of graphite-like carbon as a barrier.

Melamine is also an important member of the halogen-free flame-retardant system, and it can be used alone or in combination with other flame retardants [60,61]. Nabipour et al. [62] applied isoreticular-MOF-3 to fire safety for the first time and obtained novel flame retardants by covalent hybridization with melamine. The addition of 2% heterocyanide to EP resulted in an LOI level up to 30.5% and decreases in pHRR, THR, and SPR by 74.0%, 71.4%, and 35.6%, respectively. By CCT and residual char analysis, they analyzed the flame-retardant smoke suppression mechanism from the condensed phase and gas phase, respectively. On the one hand, the ZnO generated by MOF-3 would enhance the thermal stability of the residual char while catalyzing the formation of char residues. On the other hand, melamine could generate non-combustible gas to dilute the combustible gas concentration and produce melam and melem to improve the heat resistance of char yield.

### 3.2. DOPO and MOFs as Flame Retardants

**Figure 6:**
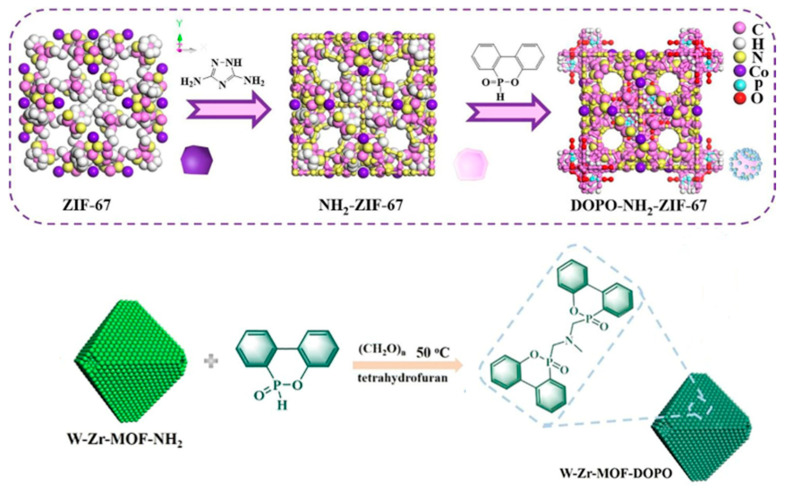
Schematic diagram of DOPO-NH_2_-ZIF-67 and W-Zr-MOF-DOPO structures. Reprinted with permission from Ref. [68]. Copyright 2021 Elsevier, and Ref. [72]. Copyright 2022 Elsevier.

9,10dihydro-9-oxa-10-phosphaphenanthrene-10-oxide (DOPO) is a phosphorus-containing flame-retardant intermediate that contains P-H bonds in its structure and has good activity against olefins, epoxy bonds, and carbonyl groups, and can react to generate a variety of derivatives [63,64]. The thermal stability of both DOPO and its derivatives is higher compared to uncycled organophosphate esters because of the benzene and phenanthrene ring structures in DOPO and its derivatives. Meanwhile, the modification of MOFs by DOPO can retain the original structure well, as shown in Figure 6. Some studies have shown that DOPO gives good flame-retardant performance, but the smoke suppression improvement is low [65,66,67]. Wang et al. [68] modified the synthesized bimetallic organic backbone (W-Zr-MOF) using DOPO to introduce a large amount of P elements while retaining the hollow octahedral morphology. The porous graded structure of the modified MOFs was used to efficiently absorb the released smoke and volatile gases, and it was able to significantly suppress the smoke and heat release. At the same time, the pyrolysis of DOPO produced a large amount of polyphosphate, which promoted the carbonization and dehydration of EP, thus inducing the formation of a dense and phosphorus-rich carbon layer. The TSP and pHRR were reduced by 36% and 58%, respectively, when 3 wt% of W-Zr-MOF-DOPO was added, and the residual char integrity was much higher than that of pure EP, as evidenced by visual inspection alone, a result also confirmed by Raman spectroscopy. It is shown that the multiple coordinated effects of multi-level pores, metals, and P elements improve the fire safety of EP composites. Liu et al. [69] used a derivative of DOPO (DMZ) to modify ZIF-8 to obtain dZIF-8, which was added to EP as a flame retardant. The dZIF-8EP composites containing 2 wt% of dZIF-8 reduced the total smoke release rate (TSRR) and maximum smoke density by 47% and 36%, respectively, compared to pure EP. It was shown that MOFs and DOPO have synergistic effects to enhance the smoke suppression performance of the polymer. In addition to the graft modification of DOPO on the original MOFs, some MOFs were coated on DOPO [70,71]. The metal elements in MOFs were also used to promote catalytic carbonization, forming a denser char layer and providing a more effective barrier effect.

### 3.3. Phosphonitrile Compounds and MOFs as Flame Retardants

Phosphonitrile polymers are a class of inorganic–organic polymers with alternating P and N double bonds as the main chain structure, which can be generally divided into two categories: cyclic polyphosphonitrile and linear polyphosphonitrile. Among the cyclic polyphosphonitrile, cyclotriphosphonitrile and cyclotetrakisphosphonitrile are the most common. Hexachlorocyclotriphosphonitrile (HCCP) is a very important intermediate and the most basic compound in phosphonitrile chemistry, and it plays a pivotal role in the development of phosphonitrile chemistry. Polyphosphonitrile compounds have outstanding light stability, thermal stability, good resistance to high and low temperatures, and high LOI value [73,74,75]. The main chain of polyphosphonitrile molecules is structurally stable and has a very high heat resistance temperature. What determines the physicochemical properties of polyphosphonitrile compounds is the nature and number of side chain species of phosphorus atoms in the phosphonitrile. Polyphosphazene has excellent flame-retardant applications because of its low smoke emissions and low toxicity of the gases emitted during combustion [76]. Polyphosphazene has similar customizable properties to MOFs, so when combined with MOFs, it is possible to customize a wider variety of structures with MOFs as well as to satisfy the need to avoid a single flame-retardant function. On this basis, the MOFs can be further modified by combining them with other materials to form a multi-synergistic flame-retardant effect [77,78].

According to researchers, MOFs and phosphonitrile compounds are mostly combined in the form of encapsulation or surface modification. Xu et al. [79] synthesized a Co-based MOF (MOF-71-NH_2_) and obtained organic–inorganic hybridization products by modification with phosphonitrile trimer (PCT). PS composites doped with PCT only, MOF-71-NH_2_, and PCT@MOF-71-NH_2_ were compared with pure PS using 3 wt% as a reference. With the synergistic effect of MOF and PCT, both heat release and smoke release were lower than adding only one of them, and THR and pSPR were reduced by 31.7% and 41%, respectively, compared to pure PS. Meanwhile, the increased yield and denseness of the residual char could be clearly seen by digital photographs and SEM of the residual char. Some CCT data for MOFs and phosphonitrile compounds as flame retardants are shown in Table 2. The selected flame-retardant additions are all 3%, and the polymer matrix is mostly EP, which is convenient as a comparison. It can be seen from the table that there are large differences in the degree of decrease of THR and TSP, compared to the pure resin matrix part of the composite, which is not significantly decreased, probably because of the different catalytic ability shown by different MOFs. This is also in response to the need for different matrixes to take different kinds of MOF composite attempts to compare. Among them, the decrease of Fe-MOF@PZS as a flame retardant is particularly significant, which is mainly attributed to the Fe_2_O_3_ and phosphide produced during the combustion process, and the char formed a dense and hard char ceramic layer, which effectively protected the unburned EP composites [80].

### 3.4. Other Phosphorus and Nitrogen Compounds and MOFs as Flame Retardants

**Figure 7:**
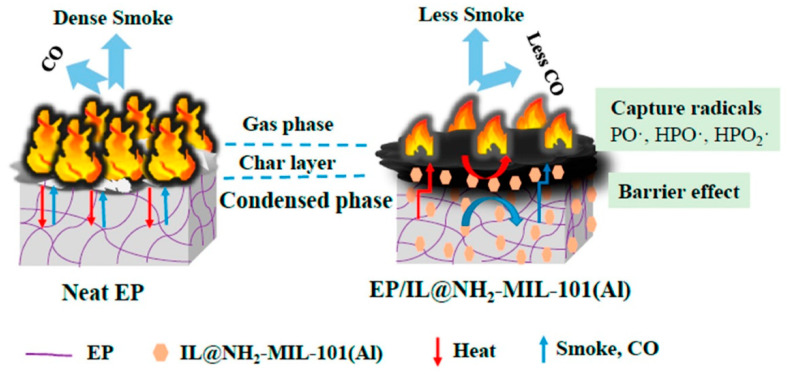
Schematic illustration of the proposed mechanism of EP/IL@NH_2_-MIL-101(Al) [89].

In addition to the above-mentioned phosphine and nitrogen compounds that are used more synergistically with MOFs, there are some others that have been explored by researchers and have shown good results, such as phosphonates [85], α-zirconium phosphate [86], and polyaniline [87]. Some of the organic ligands in MOFs contain -NH_2_. On the one hand, -NH_2_ helps to increase the compatibility with EP. On the other hand, due to the presence of -NH_2_, NH_3_ is released during the combustion of EP, which dilutes the combustible gas and acts as a flame retardant in the gas phase. Wang et al. [88] modified NH_2_-MIL-101(Al) with polyethyleneimine (PEI) and used adsorbed dye as a further modification step to achieve sustainable application of MOF materials. Meanwhile, the synergistic effect of adsorbed dyes, MOF, and PEI enhanced the flame-retardant properties of EP. An ionic liquid (IL) containing phosphorus and nitrogen elements was synthesized, and the composite was obtained with NH_2_-MIL-101(Al) [89]. Combining the respective flame-retardant characteristics of both, the advantages are exerted in both the condensed phase and the gas phase, as shown in Figure 7. In addition, some researchers have constructed lamellar structures containing phosphorus and nitrogen elements and combined or modified them with MOFs in the form of two-dimensional/three-dimensional structures to obtain flame-retardant composites with fire and smoke suppression [90,91]. Zhang et al. [92] designed flame-retardant hybrids with specific functions using UiO-66 as the core and Prussian blue analogues (PBA) and poly(dopamine) (PDA) as custom components of the laminate structure. The composite of EP/UiO66-PDA-PBA-3 showed by CTT results that pHRR and CO production (COP) were reduced by 50% and 66%, respectively, and the residual char increased from 10.6% to 19.8%. This indicates that the constructed layered structure could be used as a new method of effective fire protection.

## 4. Carbon-Based Materials and MOFs as Flame Retardants

The development of carbon-based materials has a long history, as carbon is an element that is very commonly found in nature. Because carbon atoms can be bonded into numerous isomers of molecules and atomic-type crystals, they can be stacked and aggregated in different ways and orientations at the micron and nanometer scales, forming a wide variety of weaves. Carbon as a single element can form many substances with completely different structures and properties. With the advancement of characterization techniques in recent years, fullerenes (C_60_) [93], carbon nanotubes (CNTs) [94], and graphene [95] have been discovered one after another, and since then a new chapter of exploring carbon nanomaterials has been opened. Researchers have modified carbon materials at the microscopic and mesoscopic scales to prepare carbon materials with various novel functions for applications such as wave absorption [96,97,98], medical treatment [99], electrochemical supercapacitors [100,101], catalysis [102], and flame retardancy [103]. Carbon materials mostly have high thermal stability and thermal conductivity and partially enhance the mechanical properties of the polymer matrix. On the one hand, the carbon material itself acts as a physical barrier and provides a barrier effect. On the other hand, it contributes to the formation of a dense protective layer and helps to insulate heat and combustible gases.

### 4.1. Graphene and MOFs as Flame Retardants

**Figure 8:**
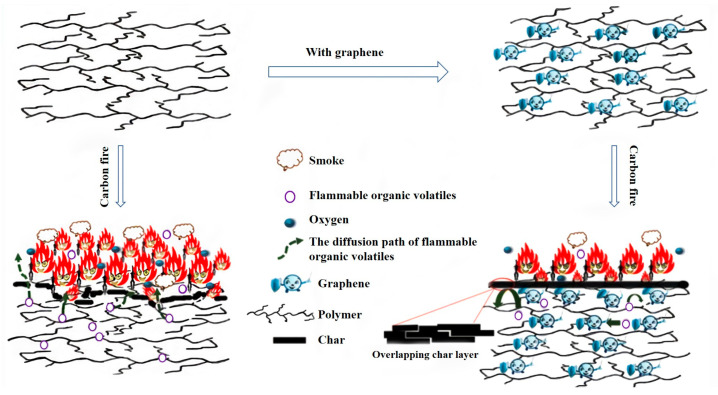
Schematic diagram showing the flame retarding mechanism of graphene-based flame retardant under ideal conditionReprinted with permission from Ref. [107]. Copyright 2016, Springer Nature.

Graphene is a two-dimensional material composed of a single layer of carbon atoms that possesses a great specific surface area and exhibits excellent thermal, electrical, and mechanical properties. Both graphene and its derivatives have good flame-retardant potential [104,105]. The flame-retardant mechanism based on graphene is vividly illustrated in Figure 8. However, due to the strong van der Waals and π-π gravitational forces between the graphene nanosheets, it is easy to agglomerate in the polymer matrix and difficult to disperse uniformly, resulting in the flam-retardant effect being affected [106]. The modification functionalization required by this problem associates the advantages of MOFs.

To improve flame retardancy by solving agglomeration problems, Hou et al. [108] used ZIFs as templates for layered double hydroxides (LDHs) while allowing in situ synthesis on the surface of graphene oxide (GO) or CNTs, which directly inhibited the agglomeration behavior of carbon materials. Compared with pristine GOs or CNTs, the composites combined with ZIF-derived hybrids exhibited extraordinary flame-retardant properties in unsaturated polyester resin (UPR). In addition, they were the first group to combine GO with MOF as a flame retardant for PS. By combining with GO, the agglomeration of vertically grown Ni-MOF sheets was significantly suppressed, and the resulting GOF hybrids had a large specific surface area and pore volume, which promoted the adsorption of volatile pyrolysis products. In studying the release of toxic gases during combustion, a steady-state tube furnace (SSTF) was used to evaluate the CO release. The COP of the PS/GOF composite was reduced by 52.3% compared to pure PS. Unlike agglomerated rGO sheets and NiO particles, the nanoscale NiO on the rGO surface provides a larger surface area and more active sites for the catalytic reduction of CO, thus reducing the COP of the composite [109]. The combustion data of graphene alone as a flame retardant and in combination with MOFs as a flame retardant are shown in Table 3. It is easy to see that when only graphene material is added as a flame retardant, the flame-retardant performance has improved compared with the pure polymer material. However, after compounding with MOFs, the flame-retardant data were further improved on this basis, which is sufficient to show that the combination of MOFs and graphene materials is the right choice to explore the improvement of flame-retardant performance.

**Figure 9:**
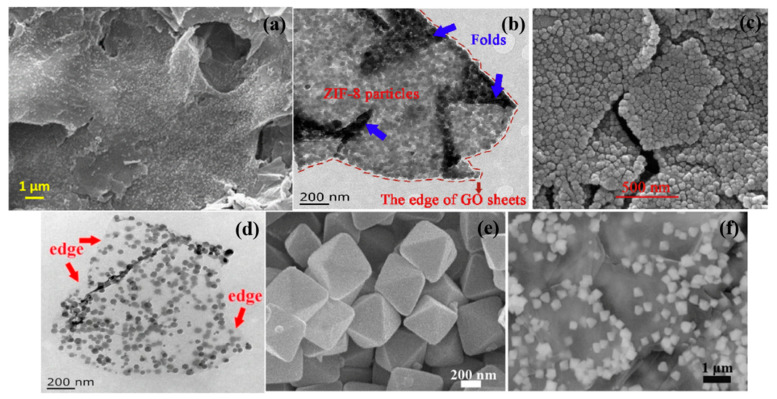
(**a**) SEM image of Co/Zn-MOF@GO nano-hybrids with big crystal distributed; (**b**,**c**) TEM and SEM of GPZ; (**d**) TEM of nano-ZIF-8@GO; (**e**,**f**) SEM image of UiO-66-NH_2_, TEM image of the UiO-66-NH_2_/GnPs. Reprinted with permission from Ref. [78]. Copyright 2022 Elsevier, Ref [115]. Copyright 2020 Elsevier, Ref. [116]. Copyright 2020 Elsevier, and Ref. [117]. Copyright 2018 Springer Nature.

Because graphene is a sheet structure with large surface area and abundant functional groups, most MOFs combined with it are grown directly on the surface of the sheet. It can be clearly seen from Figure 9 that MOF nanoparticles grow uniformly on the surface, and MOF nanoparticles can better catalyze the carbonization while the graphene sheet acts as barrier. In addition, it can be combined with other materials for modification and grafting to build ternary or even more multi-component flame-retardant materials. Xu et al. [111] doubly modified RGO with LDH and ZIF-67, and the flake LDH improved the RGO stacking phenomenon. The test results showed that RGO-LDH/ZIF-67 could effectively improve the smoke suppression effect, and the maximum smoke density (D_s,max_) was reduced by 56.2% compared with pure EP, and the graphitization of residual carbon was significantly improved. Xu et al. [113] also synthesized leaf-type Co-ZIF-modified graphene and incorporated it into TPU together with conventional flame retardants. The thermogravimetric-infrared (TG-IR) test showed a significant reduction in the release of CO and HCN, etc. compared to pure TPU. The pHRR, THR, and TSP of TPU composites were significantly reduced by 84.4%, 70.1%, and 62.5%, respectively. On the one hand, the metallic cobalt provided by MOFs facilitates catalytic carbonization. On the other hand, the flake RGO and the catalytically formed carbon together play a physical barrier role.

### 4.2. Other Carbon-Based Materials and MOFs as Flame Retardants

**Figure 10:**
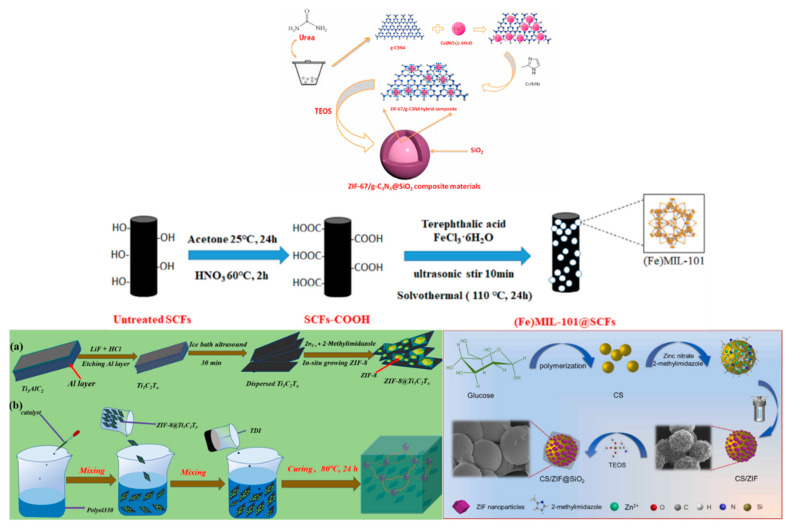
Scheme of MOFs loading on carbon-based materials. Reprinted with permission from Ref. [121]. Copyright 2021 Elsevier, Ref. [122]. Copyright 2022 Elsevier, Ref. [123]. Copyright 2022 Elsevier, and Ref. [125]. Copyright 2021 John Wiley & Sons Ltd.

In addition to graphene, researchers have also combined other carbon-based materials with MOFs in order to investigate the effect of different carbon-based materials and MOFs exerting synergistic effects on flame-retardant properties. Some design options of MOF loading on carbon-based materials, such as electrostatic interaction, hydrothermal reaction, and in situ growth, are provided in Figure 10, which can provide ideas for future modification designs. Graphite carbon nitride (g-C_3_N_4_) as an emerging two-dimensional material with a unique electronic structure, thermal stability, and high nitrogen content has some advantages for flame-retardant applications [118,119]. Chen et al. [120] constructed MIL-53 (Fe)@C/g-C_3_N_4_ by hydrothermal reaction and added it to UPR. The pHRR, pSPR, and CO_2_Y of the UPR composites decreased by 39.8%, 33.3%, and 37.3% compared to the pure UPR when the addition amount was 4%. The addition of MOFs helped catalyze the formation of stable carbon layers more than the UPR composites with the addition of g-C_3_N_4_ alone, and the suppression of smoke was significantly enhanced. Xu et al. [121] successfully prepared ZIF-67/g-C_3_N_4_@SiO_2_ hybrids and enhanced the flame-retardant properties of EP. ZIF-67 generated cobalt oxides during combustion, and the smoke index decreased significantly due to the large specific surface area that could adsorb smoke and volatile particles. Compared with pure EP, the pores of residual char became smoother and denser after combustion, and the I_D_/I_G_ values from Raman spectra (I_D_ and I_G_ represent the D peak of amorphous carbon and G peak of crystalline graphitic carbon, respectively) indicated the enhancement of graphitization. The assembly of MXenes material (Ti_3_C_2_T_x_) and the combination of carbon spheres (CS) and MOFs can also be seen in Figure 10. The advantages of being two-dimensional and three-dimensional carbon-based materials for the barrier of combustible gases, heat, and smoke, coupled with the catalytic effect of MOFs, exhibit the synergistic effect of both to enhance the flame-retardant properties of the polymer [122,123,124].

### 4.3. MOFs and Biomass Materials as Flame Retardants

Able to fit the concept of a green environment, biomass as a flame retardant has become a research hotspot. Because biomass as a biological resource is a renewable resource, it is not toxic and is also in line with the concept of sustainable development. Biomass as a flame retardant is broadly available in proteins, amino acids, oils, and sugars. The reason why bio-based flame retardant is included in this part of carbon-based materials is to consider that biomass and carbon are inseparable. Some biomasses can be used as a carbon source in flame retardants, and there are other biomasses that can promote carbon formation during combustion [126,127]. Among them, the modification of MOFs with phosphorus-rich phytic acid (PA) not only regulates the degradation of polymers but also promotes the cross-linking of EP combustion decomposition products into carbon. β-cyclodextrin (β-CD) is a natural source of carbon, and Zhang et al. [128] functionalized MOFs with PA and formed layered hybrids with β-CD to be added to EP as flame retardants. pHRR and SPR were reduced by 41% and 62%, respectively. The modified MOFs can act as both an acid and gas source and can promote more carbon production from β-CD, enhancing co-efficient flame retardancy. The combination of naturally derived cellulose and MOFs to construct gas barrier gels is not only economical and environmentally friendly, but also has a clear cross-linked network structure [129,130]. It also has certain adsorption abilities due to the mesh structure and great specific surface area, which can improve the handling of gaseous products by MOFs during the combustion process.

For the combination of biomass and MOFs, the structure is not limited to simple encapsulation and growth, but to build a multifaceted composite structure with more flame-retardant elements [61,114,131]. In terms of application, it is not only limited to flame retardants, but also to increase mechanical properties, adsorption applications, and hydrophobicity, etc. [132,133]. Xie et al. [134] used direct melt blending of dry wine lees, ZIF-8 and PP, and the mechanical properties of the obtained composites were significantly improved, with tensile strength and strain at break values of 34.2 MPa and 24.8%, respectively. This was due to the introduction of ZIF-8 as an interfacial modifier and energy-absorbing nanocrystals. In addition, the LOI value reached 25.0% at the ZIF-8 addition of 3 wt%, and the residual carbon not only became smooth and dense but also had a significant increase.

## 5. MOFs as Derivatize Templates and Hybrids with LDHs as Flame Retardants

In recent years, due to the rise of MOFs, their derivatives have been increasingly discovered and studied. The properties and advantages of MOFs have been mentioned above, because these structural properties can be preserved in derived materials, which can have a wider scope of application if they are suitably tuned (morphology, porosity size, and crystal size, etc.). Moreover, MOFs have been demonstrated as highly versatile precursors capable of deriving a wide variety of nanostructures, including carbon materials, metal oxides, metal phosphides, metal carbides, and LDHs [135,136]. These MOFs-derived materials are often used in batteries [137], supercapacitors [138], electrocatalysis, photocatalysis [139], and sensing, etc. Flame-retardant applications are relatively rare, and LDHs are mostly used for flame-retardant applications at this stage.

### 5.1. MOFs as Templates to Derivatize LDHs as Flame Retardants

LDH is a nano-filler, which combines the characteristics of traditional metal oxides and layered silicates and is widely used as a halogen-free flame retardant. On the one hand, LDHs with abundant oxygen-containing groups are well dispersed in polymers. On the other hand, the H_2_O decomposed by LDHs during combustion is beneficial to reduce the heat release rate, and the transition metal elements contained can generate metal oxides to promote the catalytic effect [140]. Li et al. [141] anchored ZIFs on LDH as flame retardants mixed into EP and made a comparison between ZIF-8 and ZIF-67 regarding flame-retardant properties. While showing the good flame-retardant performance of LDH, it could be presumed that the derivation of LDH from MOFs will have better results. MOFs have been shown to be ideal templates for the preparation of LDHs, and ZIFs can be used as precursors for the construction of different LDHs on a variety of materials [142,143]. Pan et al. [144] constructed a sandwich-type three-dimensional hybrid structure using ZIF-67 by growing NiCo-LDH in situ on both sides of graphene nanosheets, which avoided agglomeration on the graphene nanosheets and provided high specific surface area. The pHRR and TSP after addition to EP showed a significant decrease compared to pure EP, and the residual char amount increased from 11.9% to 21.6%. It was easy to see from the digital photos of each residue that rGO had poor fire performance due to stacking agglomeration, which also indicated that the catalytic effect of nickel-iron ions in LDH promoted the formation of carbon layers, thus reducing the heat and mass transfer in the gas and solid phases. Hou et al. [108] adjusted the Zn/Co ratio of bimetallic ZIF, which would keep the original structure better and be beneficial to the growth and arrangement of derivatives on the carrier. Sandwich structures and necklace structures were constructed using GOs and CNTs as carriers, respectively. UPR/GO@LDHs and UPR/CNTs@LDHs had a 27.4% and 24.6% reduction in THR and 46.1% and 33.9% reduction in TCO compared to pure UPR. In addition to 3D multilayer structures [145], there were LDHs grown on hollow nanomicrospheres [146], loaded on different carriers with flower-like structures derived from MOFs [147]. The variety of forms was to make the hybrids more functionalized and introduced more elements or groups. The multilayer structure helped physical barrier and hierarchical progressive catalysis, and the hollow microspheres were surface-modified to facilitate charring at the filler-substrate interface. While discussing LDHs as flame retardants, attention was also paid to their effects on the mechanical properties of polymers. Some data of pure polymer matrix and composites are listed in Table 4. It can be seen in the table that, after the addition of flame retardant, the tensile strength of each composite basically shows a trend of strengthening, which also confirms the good dispersion of the derived LDH flame retardant in the matrix.

### 5.2. MOFs as a Template for Other Derivatives as Flame Retardants

**Figure 11:**
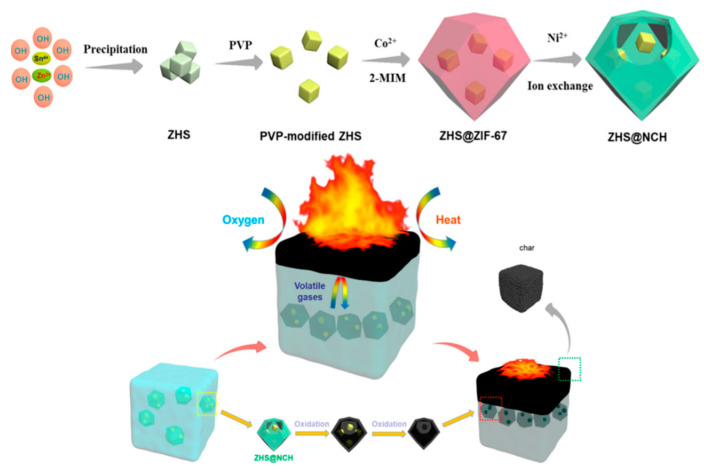
Schematic illustration for preparation process of ZHS@NCH and Schematic illustration of flame-retardant mechanism for ZHS@NCH. Reprinted with permission from Ref. [152]. Copyright 2019, American Chemical Society.

In addition to serving as templates for the derivation of LDHs, MOFs also have other derivatives added as flame retardants. Zhang et al. [152] proposed the concept of “secondary agglomeration” based on carriers and loadings, which was further investigated based on the ease of aggregation of nanoparticles in polymers. To solve this problem, zinc hydroxystannate (ZHS) nanoparticles were encapsulated by ZIF-67 and then layered Ni-Co hydroxides nanocages (NCH) were obtained using ZIF-67 as a template. Due to the confinement of ZHS by the egg yolk shell-like structure formed by NCH, the possible detachment of ZHS from the carrier due to weak loading force was avoided. In order to compare the performance differences, flame retardant tests of ZHS@NCH, ZHS, and NCH were carried out, respectively. Figure 11 shows the process of ZHS@NCH preparation and the flame-retardant mechanism. Both the external NCH and the internal ZHS could catalyze the carbonization of EP, thus exempting the lower substrate from further combustion. There was also a simple hydrothermal reaction using Ni-MOF as a template to introduce P elements, and the surface was composed of microspheres while retaining the original nanorod morphology. CCT showed that both THR and TSP are reduced, and the mechanical properties are improved [153]. In addition, there are studies to introduce more metallic elements or flame-retardant elements, and some other properties will be taken into account while enhancing the flame-retardant properties [154,155]. It is suggested that the use of MOFs as derivatives to combine other hybrids, in connection with many other application scenarios, broadens the limits of single use.

## 6. Conclusions and Future Perspectives

In conclusion, MOF-based flame retardants, as a material developed in recent years, can take the responsibility of flame-retardant applications for polymers. Through the structural analysis of MOFs, both organic ligands and metal sites have certain flame-retardant functions, but the specific question of which plays a greater role is inconclusive. There are also large differences in the role of metal oxides formed by different transition metals in the combustion process to play a catalytic carbonization of different polymers, and a large number of comparisons are still needed to facilitate further analysis. Aggregation and agglomeration of nanomaterials is a major issue, and the organic ligands of MOFs have the function of promoting compatibility with polymers, and the morphologies of the original MOFs used at this stage are regular and ordered. However, what type of ligand is more effective is still inconclusive, but this can be solved by introducing other conventional materials as carriers.

At this stage, there is limited research on MOFs as flame retardants directly, and the inorganic–organic characteristics of MOFs themselves are used to construct multi-faceted flame-retardant systems. By introducing inorganic flame-retardant elements and transition metal elements, MOFs are modified to introduce organic flame-retardant groups to construct different core-shell structures, three-dimensional multilayer structures, and sheet carrier structures, etc. All of them are designed to take advantage of the synergistic effect of components or structural advantages on the basis of the original ones, and to carry out flame-retardant functions from the gas phase and the condensed phase, respectively.

More attempts are needed to see if new MOFs can be applied to flame-retardant polymers in the future, and research on existing MOFs can be combined with other new materials containing flame-retardant elements or moieties. The combination of biomass materials and MOFs is still in the initial stage, and the construction of green flame-retardant systems could enhance the combination of both. Although some types of MOFs are now commercially available, the price is not enough to be used in flame retardants. Moreover, a single MOF does not meet our expectation of flame-retardant effect, and further modification is needed. Therefore, we believe that the future commercialization of MOF flame retardants requires the following conditions: (1) The need for innovative approaches to the production of large quantities of MOFs. (2) The search for materials that synergistically demonstrate excellent flame-retardant effect with MOFs, and at the same time this material should show the advantage of reasonable price. (3) Avoid overly complex functionalization of MOFs.

## Figures and Tables

**Table 1:** Data comparison of MOF/APP as flame retardant and pure matrix.

Samples	THR	pHRR	TSP	pSPR *	Residues	Reference
	MJ/m^2^	kW/m^2^	m^2^	m^2^/s	%	
TPU	78	1359	10.3	0.11	0.1	[23]
TPU/6.0APP	65	268	8	0.06	11.0	
TPU/4.5APP/1.5Co-MOF	63	257	7.1	0.04	15.2	
TPU	138.9	909.6	-	-	1.74	[55]
TPU/APP	117.0	270.7	-	-	24.16	
TPU/APP/Cu_0_._0625_	42.6	218.7	-	-	28.19	
PLA	81.7	450.8	2.6	-	0.1	[57]
PLA-5.0APP	70.6	396.6	2.4	-	4.1	
PLA-3.3APP/1.7Ni-MOF	65.9	329.6	1.3	-	5.4	
Blank	100.82	1925	52.11	0.56	-	[58]
Intu/Neat	41.72	738	43.69	0.44	-	
Intu/MOF	31.92	522	35.33	0.35	-	
EP	105	1254	31	0.38	8.0	[59]
5APP/EP	103	924	31	0.26	17.4	
(ZIF-67+APP)/EP	74	495	27	0.16	23.8	

* Peak of smoke production rate.

**Table 2:** Data on MOFs and phosphonitrile hybrids as flame retardants.

Samples	THR	pHRR	TSP	pSPR	Residues	Reference
	MJ/m^2^	kW/m^2^	m^2^	m^2^/s	%	
EP	88.85	1631.2	-	0.347	-	[54]
EP/ZIF-8@PZN-3	48.53	819.2	-	0.319	-	
EP	92.5	1073	24.83	0.334	16.2	[80]
EP/3 Fe-MOF@PZS	58.4	749	14.55	0.212	28.5	
EP	116.7	1408.9	36.7	0.43	2.7	[81]
EP/ZIF-8@HCCP-ZP-50	100.7	745.3	28.7	0.26	15.87	
EP	106.65	1601	30.94	-	0.52	[82]
3% Salen-PZN-Cu@Ni-MOF/EP	95.17	1114	28.84	-	3.55	
PUA	108.37	935.4	-	-	1.13	[83]
PUA/ZnO@MOF@PZS 3.0	87.13	670.6	-	-	5.99	
EP	88.1	1127	35.6	-	1.8	[84]
EP/ZIF@PZS	75.5	913	28.1	-	8.7	

**Table 3:** Data on graphene and its compounding with MOFs as flame retardants.

Samples	THR	pHRR	TSP	SPR	Residues	Reference
	MJ/m^2^	kW/m^2^	m^2^	m^2^/s	%	
EP	41.2	940	63.0	0.82	-	[106]
RGO/EP	31.9	473	56.4	0.72	-	
ZIF-8/RGO/EP	27.3	332	36.8	0.53	-	
EP	57.2	1212	59	0.86	9.9	[110]
EP/RGO-2	54.5	830	49.4	0.64	12.7	
EP/ZIF-67/RGO-B-2	33.5	423	32.7	0.37	20.7	
EP	58.6	1355	59.0	0.78	10.0	[111]
EP/RGO-LDH	43.3	580	34.3	0.52	15.7	
EP/RGO-LDH/ZIF-67	37.9	464	29.9	0.38	17.9	
EP	-	992	36.9	-	16.6	[112]
EP/0.5GO-9.5IFR	-	812	23.3	-	-	
EP/0.5MOF@GO-9.5IFR	-	532	18.3	-	33.1	
TPU	192.7	1573	16.0	-	9.2	[113]
TPU/IFR/RGO	87.8	422	10.4	-	18	
TPU/IFR/Co-ZIF-L/RGO	57.6	245	6.0	-	18.4	
EP	96.9	1133	-	-	17.9	[114]
EP/GO	91.1	919	-	-	20.2	
EP/ZIF@GO	75.4	507	-	-	24.0	

**Table 4:** Data on MOFs-derived LDHs as flame retardants.

Samples	THR	pHRR	TSP	TS *	Residues	Reference
	MJ/m^2^	kW/m^2^	m^2^	MPa	%	
EP	96	961	30	39.7	11.9	[144]
EP/2% rGO@LDH	80	327	21	45.3	21.6	
EP	88.9	1235	24.1	59.6	-	[146]
EP/HGM@LDH@DOPO	76.7	539	22.9	48.4	-	
EP	72.9	1361	26.4	ANE **	4.9	[148]
EP/2.5% MgAl@NiCo	44.7	455	19.8		10.9	
EP	44.6	426.9	-	-	7.1	[149]
EP-4.0	39.6	294.9	-	-	11.2	
FPUF	33.8	-	1.74	71.2	-	[150]
6 wt% Ti_3_C_2_Tx@MOF-LDH	29.5	-	1.46	77.1	-	
PUA	108.4	935.4	14.1	23.72	6.9	[151]
PUA/NiMoO_4_@Co-Ni LDH3.0	94.6	613.5	11	25.63	13.5	

* Tensile strength, ** almost no effect.

## Data Availability

Not applicable.
